# New AMH cutoff values for warning of decreased ovarian response based on MCL characteristics in young women: a retrospective study using a propensity score-matching analysis

**DOI:** 10.1186/s12884-022-05294-7

**Published:** 2022-12-24

**Authors:** Jia-Bei Lv, Ying Han, Xin-Yan Wang, Li-Na Yuan, Jun-Rong Diao, Ya-Zhen Fan, Hai-Ning Luo

**Affiliations:** 1 Tianjin Medical University, Tianjin, China; 2 Tianjin Key Laboratory of Human Development and Reproductive Regulation, Tianjin Central Hospital of Obstetrics and Gynecology/Nankai University Afliated Maternity Hospital, No 156 Sanma Road, Nankai District, Tianjin, 300100 China

**Keywords:** AMH, Menstrual cycle length, Ovarian response, Granulosa cells, Oocytes retrieved

## Abstract

**Background:**

Menstrual cycle length (MCL) and ovarian response varies widely among women of childbearing age. They are provided with anti-Mu¨llerian hormone (AMH) cutoffs for “normal” and “weakened” ovarian responses, which give an early warning of the onset of decreased ovarian response.

**Methods:**

This was a retrospective study in women aged 21 to 35 years with MCLs of 21–35 days receiving in vitro fertilization (IVF) treatment at Center for Reproductive Medicine from October 2018 to October 2021. Intergroup variables were balanced using propensity score matching based on age and BMI, and each case patient (patients with MCLs of 21–25 days) was matched with three control patients (patients with MCLs of 26–35 days). A receiver operating characteristic curve was used to calculate the AMH cutoff values.

**Results:**

We included 135 patients with MCLs of 21–25 days and 405 matched control patients with MCLs of 26–35 days who received IVF treatment. The case group had significantly fewer retrieved oocytes, lower AMH values and higher initial and total Gonadotropin (Gn) levels during controlled ovarian hyperstimulation than the control group. The ovarian response began to decrease when AMH was < 3.5 ng/ml in the case group and < 2.7 ng/ml in the control group.

**Conclusion:**

In young women with MCLs of 21–35 days, short MCL was negatively correlated with AMH values and the number of oocytes retrieved. In patients with MCLs of 21–25 days and 26–35 days, the AMH cutoff values corresponding to the onset of decreased ovarian response were 3.5 ng/ml and 2.7 ng/ml, respectively.

## Introduction

Although a negative correlation between ovarian reserve and age has been widely recognized in previous literature [1], ovarian reserve varies greatly among women of the same age [2]. Additionally, we found that the length and regularity of the menstrual cycle varies among women of childbearing age. An increased menstrual cycle length (MCL) is associated with improved ovarian response to gonadotropin (Gn) stimulation, improved embryo quality and higher pregnancy and delivery rates [3]. A shorter MCL may indicate ovarian aging [4], which could differ from the rate of actual aging. Ovarian aging is an inevitable process in all women; the length of the menstrual cycle decreases with age, and such decreases often occur during the perimenopausal period in older women [5]. However, shorter MCLs also exist in some young women. Few studies have systematically examined whether menstrual cycle characteristics are directly related to indicators of reproductive health and fertility in young women who have normal MCLs [3, 6, 7].

Various markers are available to assess the ovarian function, such as serum FSH, serum oestradiol, because of their cyclic fluctuations, testing at specific times of the menstrual cycle is meaningful for assessing ovarian function, which lacks convenience for patients [8]，E2 is never used alone as a marker for the ovarian function [9] . However, AMH can be measured on any day of the menstrual cycle and can also be used alone to assess functional ovarian function. Follicle ultrasound counting may be more dependent on operator experience, So AMH is more objective than it. In addition, AMH was a coregulator of steroidogenesis in granulosa cells [10, 11], during last years, AMH has been gaining ground in the scientific literature and clinical practice. The anti-Müllerian hormone (AMH) value is a reference value that has been widely used to evaluate ovarian reserve in recent years. AMH is a member of the transforming growth factor β (TGFβ) superfamily, has a special inhibitory effect on follicular development and primordial follicle recruitment, participates in the selection of dominant follicles and plays an important role in follicular growth [12]. AMH is mainly secreted by the granulosa cells of the antral follicles in females [13], and there is no statistically significant fluctuation in AMH values throughout the menstrual cycle [14]. Granulosa cells are an important growth and differentiation marker in the complex physiological process of follicular development. Autocrine and paracrine substances in granulosa cells can promote granulosa cell proliferation and follicular growth. Interaction with oocytes is also a necessary condition for follicular development and the maintenance of their normal function [15–17]. Therefore, granulosa cells play an important role in the regulation of primordial follicle initiation, growth and development. Previous studies have proposed that AMH is associated with apoptosis of granulosa cells [18], which are closely related to ovarian reserve [19, 20], and the use of AMH values to assess ovarian reserve has been recognized.

According to Younis JS et al. [21] and some committee opinions [8], they defined the normal ovulatory cycle length and menstrual cycle length as 21–35 days. However, FIGO has previously determined that the normal frequency of menses is 24–38 days, they concluded by using 5–95% percentiles from large scale population studies among women aged 18–45 years, but this range is not completely fixed, for those aged either 18–25 or 42–45 years, the difference between the shortest and longest cycle should be 9 days or less, while for those aged 26–41 years, it is 7 days or less [22]. A cycle length of fewer than 24 days may be associated with ovulation disorders, ovarian aging and even some other ovarian diseases, however, many women with ovulatory disorders and above-mentioned conditions may also have normal-length menstrual cycles [23]. It follows that menstrual cycle length and ovulation may have some fluctuation and uncertainty, which makes it difficult to give a very definite parameter. But our study population was < 35 years old, which is slightly different from the FIGO study population, and according to our inclusion and exclusion criteria, some people with diseases have been excluded. Therefore, we finally chose to use the parameter of 21–35 days as the regular menstrual cycle, but this range varies widely in women of childbearing age. A MCL of 21–25 days is considered to be short [3, 24]. In most cases, a short MCL within the normal range is not taken seriously in young women and is not considered an indicator of reduced ovarian reserve [21]; Women younger than 35 years old failed to conceive after ≥12 months of regular, unprotected sexual，evaluation should and treatment may be initiated, but women>35 years old, only 6 months. The age of 35 is the point at which women’s fertility and ovarian response decline [8]. Age is an important factor affecting ovarian function and women with normal menstrual cycles before the age of 35 are more likely to neglect to assess fertility, to avoid these influence, we limited the age of the study population to 21 to 35 years old and the length of the menstrual cycle to 21 to 35 days to explore the relationship between these variables. Vaiarell et al. [25] concluded that because there are differences in the determination of both antral follicle count (AFC) and AMH due to laboratory errors, the number of oocytes retrieved is a more appropriate indicator to assess the ovary. Based on previous literature [26, 27], in ovarian stimulation, obtaining 15–20 oocytes is more likely to result in a better outcome, so we defined 15 oocytes retrieved as a normal ovarian response; Obtaining < 5 retrieved oocytes represent a decreased ovarian response, so we defined 5 oocytes retrieved as a poor ovarian response. In our study, the age and menstrual cycle range of the study subjects were limited to reduce the influence of these factors on the results of the study. On the one hand, Gn dosage guidance can be provided to different populations according to their MCL characteristics; On the other hand, some young women who desire to have children experience difficulties in having children because they do not notice their short menstrual cycle. In our study population, we give warning AMH cut-off values based on menstrual cycle characteristics as a reminder to assess their fertility and plan for pregnancy in a timely manner, this may avoid the further psychological, emotional and financial stress experienced in fertility treatment even the regret of lifelong infertility, which is caused by delayed pregnancy.

## Materials and methods

### Study design

This retrospective study was conducted at the Center for Reproductive Medicine from October 2018 to October 2021, through the analysis of our electronic records. Written informed consent was obtained from the participants when they presented for in vitro fertilization (IVF) treatment. The main criteria for inclusion were women aged between 21 and 35 years with an MCL between 21 and 35 days and regular menstrual cycles for whom complete data were available. The exclusion criteria were as follows: diagnosed cases of polycystic ovary syndrome; congenital gonadal dysgenesis; endocrine abnormalities affecting Gn or sex steroid secretion, such as hyperprolactinemia, thyroid dysfunction, and established diabetes, etc.; irregular menstrual cycles; secondary amenorrhea. The first exclusion step excluded 1629 patients who were not between 21 and 35 years old and 1034 patients whose menstrual cycles were not between 21 and 35 days; We also excluded 128 patients who were missing important data and 245 who met the exclusion criteria. A total of 3036 patients met the exclusion criteria. Finally, 3210 patients met the inclusion criteria, there were 136 and 3074 patients with MCLs of 21–25 days and 26–35 days, respectively. To prevent bias due to the effect of different baseline data on outcomes, PSM at a 1:3 ratio based on patient age and BMI was used. After matching, a total of 135 patients with MCLs between 21 and 25 days and 405 patients with MCLs between 26 and 35 days were finally included in the study. All patients underwent controlled ovarian stimulation (COS) using a routine protocol [long, short, gonadotrophin-releasing hormone antagonist (GnRH-a) protocol, or other protocol, including ultralong protocol, mini-stimulation protocol and natural protocol] at our center. When vaginal ultrasound suggested that the diameters of more than 3 follicles had reached 17 mm or the diameters of more than 2 follicles had reached 18 mm, follicular maturation was induced with intramuscular injection of 4000–10,000 U of human chorionic gonadotropin (HCG) or the subcutaneous injection of 0.2 mg of GnRH-a. All of the above protocols were performed under vaginal ultrasound-guided oocyte retrieval 36 h after trigger use. All transvaginal sonography (TVS) protocols and operations were performed by professional clinicians. IVF treatments were performed at our center according to routine practice. Data on hormone levels, age, body mass index (BMI), MCL, stimulation protocol and number of oocytes retrieved were collected from the medical record system.

### Menstrual cycle length

MCL was defined as number of days from the first day of bleeding. The participants were asked about their average MCL since age 16 years and whether their current MCL was regular and occurred every 21–35 days. Women who answered yes to these questions were asked to state the specific length of their cycle (21–35 days). Women with MCLs between 21 and 25 days were considered the case group, and those with MCLs between 26 and 35 days were considered the control group.

### Main outcomes

Data on hormone levels, age, BMI, MCL, stimulation protocol and number of oocytes retrieved were collected from the medical record system. IVF treatments were performed at our center according to routine practice.

### Statistical analysis

Data including age, BMI, MCL, number of oocytes retrieved, sex hormone levels, initial Gn amount and total Gn amount were collected for each patient. SPSS 23.0 software was used for the statistical analysis. The median (25th–75th percentile) was used to define continuous variables with a nonparametric distribution, and the Mann–Whitney U test and chi-square test were used for comparisons among groups. *P* < 0.05 was considered statistically significant. We performed propensity score matching (PSM) based on age and BMI and randomly selected members of the case and control groups using a 1:3 ratio for matching. We analyzed and evaluated the value of AMH to predict ovarian response by calculating appropriate receiver operating characteristic (ROC) curves and their associated areas under the curves (AUCs). The corresponding AMH cutoff was calculated according to the maximum Youden index value. The Youden index (J) was calculated using the formula sensitivity + specificity – 1. AUC values range from 0 to 1. An AUC of 0.5 indicates a low predictive value; a larger value indicates a stronger predictive value; and a value > 0.80 is considered good.

### Ethics statement

This study was approved by the Institutional Review Board (IRB) of Tianjin Central Hospital of Gynaecology Obstetrics (No: ZY2022013) and performed in accordance with the Helsinki Declaration. Written informed consent was obtained from the participants when they presented for IVF-ICSI treatment.

## Results

**Fig. 1:**
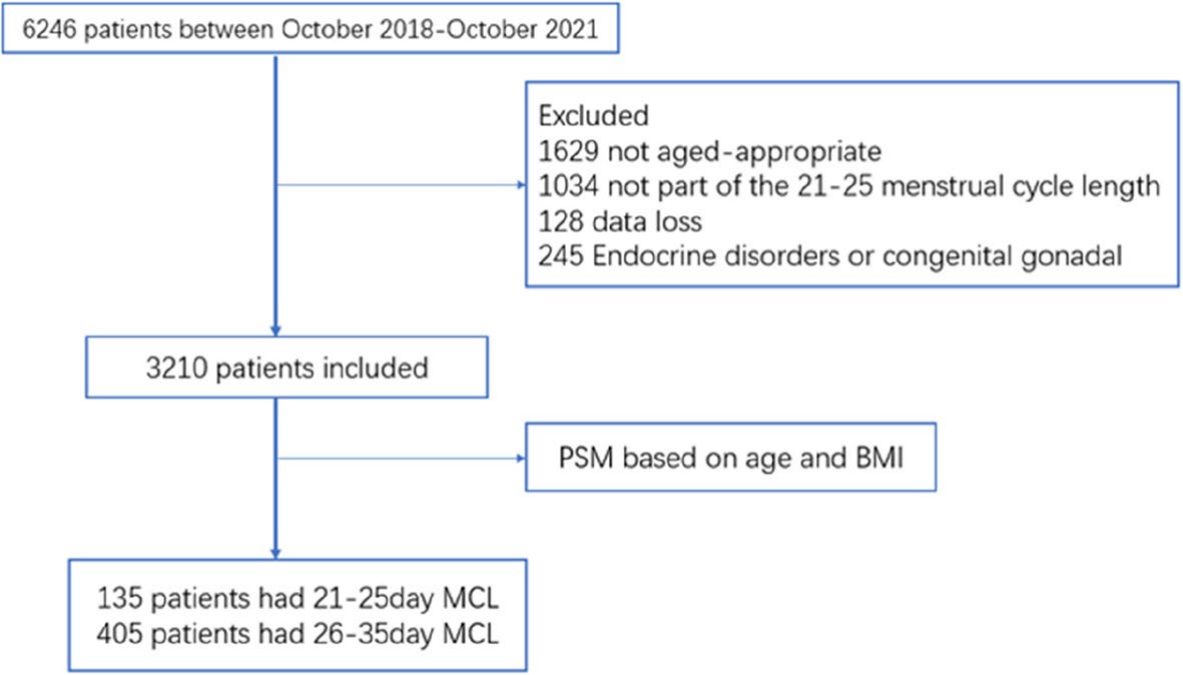
Database searching pathway and group divisions

Fig. 1 shows the selection process used to obtain the final study sample. The age and BMI characteristics of the patients in the case and control groups before and after matching are presented in Table 1. Before matching, Age level didn’t show significant difference between case and control group, but BMI level showed it, (21.53 (19.92, 23.21) vs. 22.03 (20.20, 24.41); *p* = 0.041). After matching, BMI level between case and control group was (21.56 (19.94, 23.23) vs. 21.48 (20.00, 23.55); *p* = 0.054). The baseline characteristics of the case group and the control group are shown in Table 2. The distribution of AMH significantly differed between the case group and the control group (*p* < 0.05). The AMH level showed significant difference between case and control group (2.13 ng/ml (1.20, 3.40) vs. 3.00 ng/ml (1.80, 4.30); *p* = 0.002). The LH (3.50 mIU/mI (2.50, 4.90) vs. 4.20 mIU/mI (2.90, 5.85); *p* = 0.001) and E2(47.05 pg/mL (33.60, 62.00) vs. 39.50 pg/mL (29.00, 50.55); p<0.0001) levels of the case group and the control group were statistically significant. Although the difference between case and control group of FSH (6.94 IU/L (5.46, 9.20) vs. 6.70 IU/L (5.57, 7.98); *p* = 0.122) was not statistically significant, FSH levels were higher in the control group than in the case group. The T (31.90 ng/dL (21.90, 41.40) vs. 30.30 ng/dL (22.20, 42.05); *p* = 0.759) levels of the control group and the case group were not significantly different. Compared with the control group, the case group had a significantly different number of oocytes retrieved(9(4, 16)vs. 14(9, 21); *p* < 0.001), initial amount of Gn (350 IU (300, 375) vs. 300 IU (225, 350); *p* = 0.000) and total amount of Gn (2850 IU (2400, 3300) vs. 2700 IU (2200, 3000); *p* = 0.036). Table 3 shows that when the number of oocytes retrieved was ≤5, the differences in AMH (1.19 ng/ml (0.785, 1.80) vs. 1.3 ng/ml (0.8, 1.8); *p* = 0.558), initial dosage Gn amount (350 IU (300, 375) vs. 375 IU (350, 375); *p* = 0.174) and total dosage Gn (2925 IU (2100, 3150) vs. 3000 IU (2625, 3375); *p* = 0.191) were not statistically significant. Table 4 shows the sociodemographic data of the patients, none of which was statistically significant.

**Table 1:** Baseline characteristics of case and control groups before and after matching

		Case group	Control group	*P* value
Before	Age	30.88(29.16,33.35)	31.07(29.03,32.78)	0.296
matching	BMI	21.53(19.92,23.21)	22.03(20.20,24.41)	0.045
After	Age	30.87(29.12,33.32)	31.40(29.46,33.02)	0.728
matching	BMI	21.56(19.94,23.23)	21.48(20.00,23.55)	0.474

Note: Values are presented as median (25th–75th percentile). The *p*-value is calculated using Mann–Whitney U test. Statistical significance was defined as P < 0.05

**Table 2:** Characteristics of ovarian stimulation cycles

Item	MCL groups		*P* value
	21–25 days	26–35 days	
FSH IU/L	6.94(5.46,9.20)	6.70(5.57,7.98)	0.112
LH mIU/mI	3.50(2.50,4.90)	4.20(2.90,5.85)	0.001
E2 pg/mL	47.05(33.60,62.00)	39.50(29.00,50.55)	<0.0001
T ng/dL	31.90(21.90,41.40)	30.30(22.20,42.05)	0.759
Initial amount of Gn IU	350(300,375)	300(225,350)	0.000
Total dosage of Gn IU	2850(2400,3300)	2700(2200.3000)	0.036
AMH ng/ml	2.13(1.20,3.40)	3.00(1.80.4.30)	0.002
Oocytes retrieved	9(4,16)	14(9,21)	<0.001

Note: Values are presented as median (25th–75th percentile). The *p*-value is calculated using Mann–Whitney U test. Statistical significance was defined as *P* < 0.05

**Table 3:** Characteristics of population with the number of oocytes retrieved was ≤5

Item	MCL groups		*P* value
	21–25 days	26–35 days	
Initial amount of Gn	350(300,375)	375(350.375)	0.174
Total dosage of Gn	2925(2100,3150)	3000(2625,3375)	0.191
AMH	1.19(0.785,1.80)	1.3(0.8,1.8)	0.558

Note: Values are presented as median (25th–75th percentile). The p-value is calculated using Mann–Whitney U test. Statistical significance was defined as *P* < 0.05

**Table 4:** Sociodemographic data of patients

Item	Case	Control	*P* value
Age, y	30.87(29.12,33.32)	31.40(29.46,33.02)	0.728^a^
BMI kg/m2	21.56(19.94,23.23)	21.48(20.00,23.55)	0.474^a^
Age of Marriage, y	26(24,28)	26(24,29)	0.611^a^
Age at menarche, y	13(12,13)	13(12,13)	0.059^a^
Duration of infertility	4(2,6)	4(3,5)	0.065 ^a^
Type of infertility			0.055^b^
primary infertility	48(35.6%)	172(42.5%)	
secondary Infertility	87(64.4%)	233(57.5%)	
Educational level			0.377^b^
primary School and below	11(8.1%)	20(4.9%)	
middle school	37(27.4%)	112(27.7%)	
specialized, University and above	872(64.4%)	310(67.4%)	

Note: Values are presented as median (25th–75th percentile) or n (%)

^a^*p*-value is calculated using Mann–Whitney U test

^b^*p*-value is calculated using chi-square test

Statistical significance was defined as *p* < 0.05

### ROC analysis

**Fig. 2:**
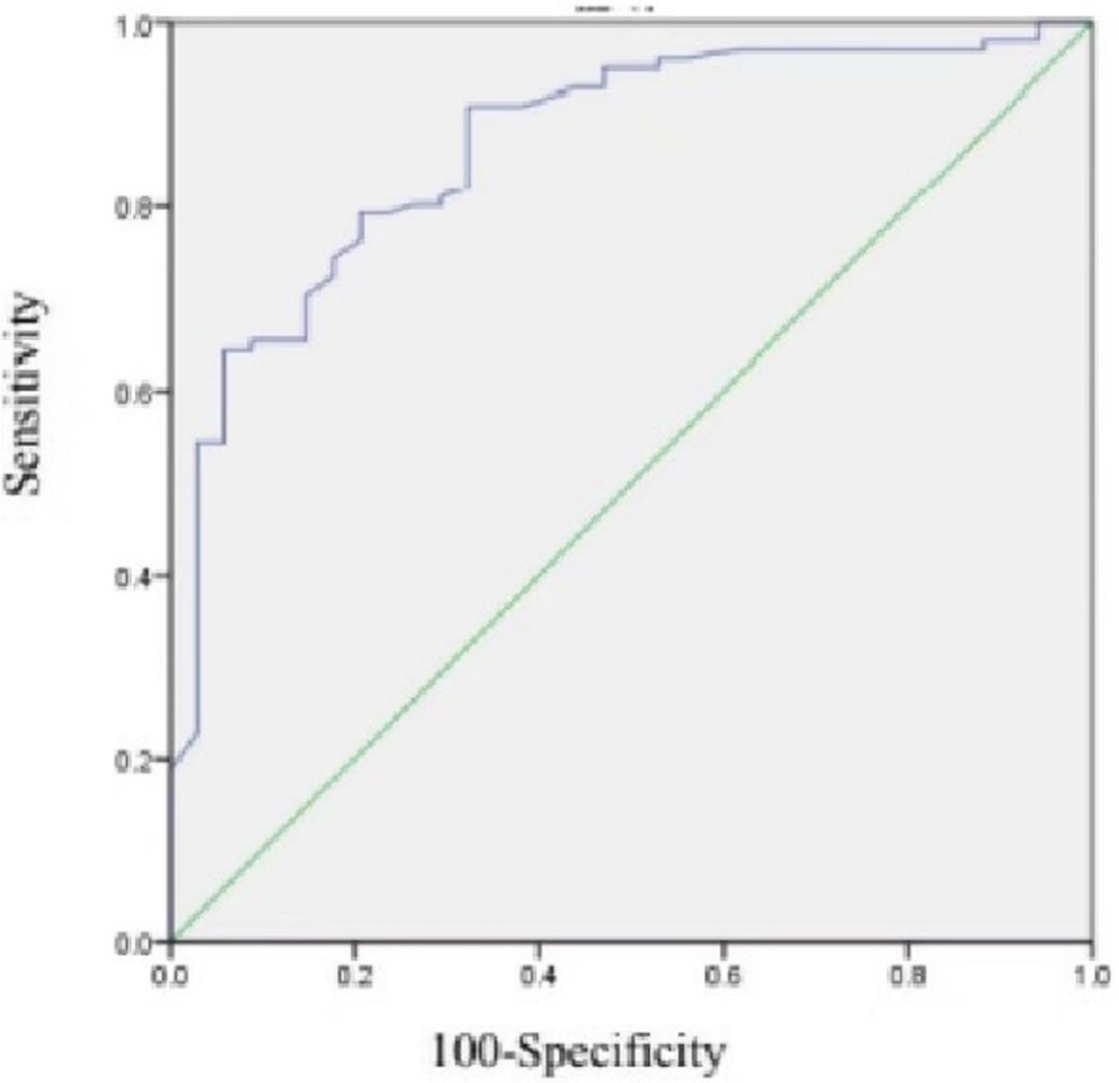
Receiver operating characteristic (ROC) curve of population with MCL of 21-25 days.The area under the TD curve is 0.866. 95% CI 0.798–0.934

**Fig. 3:**
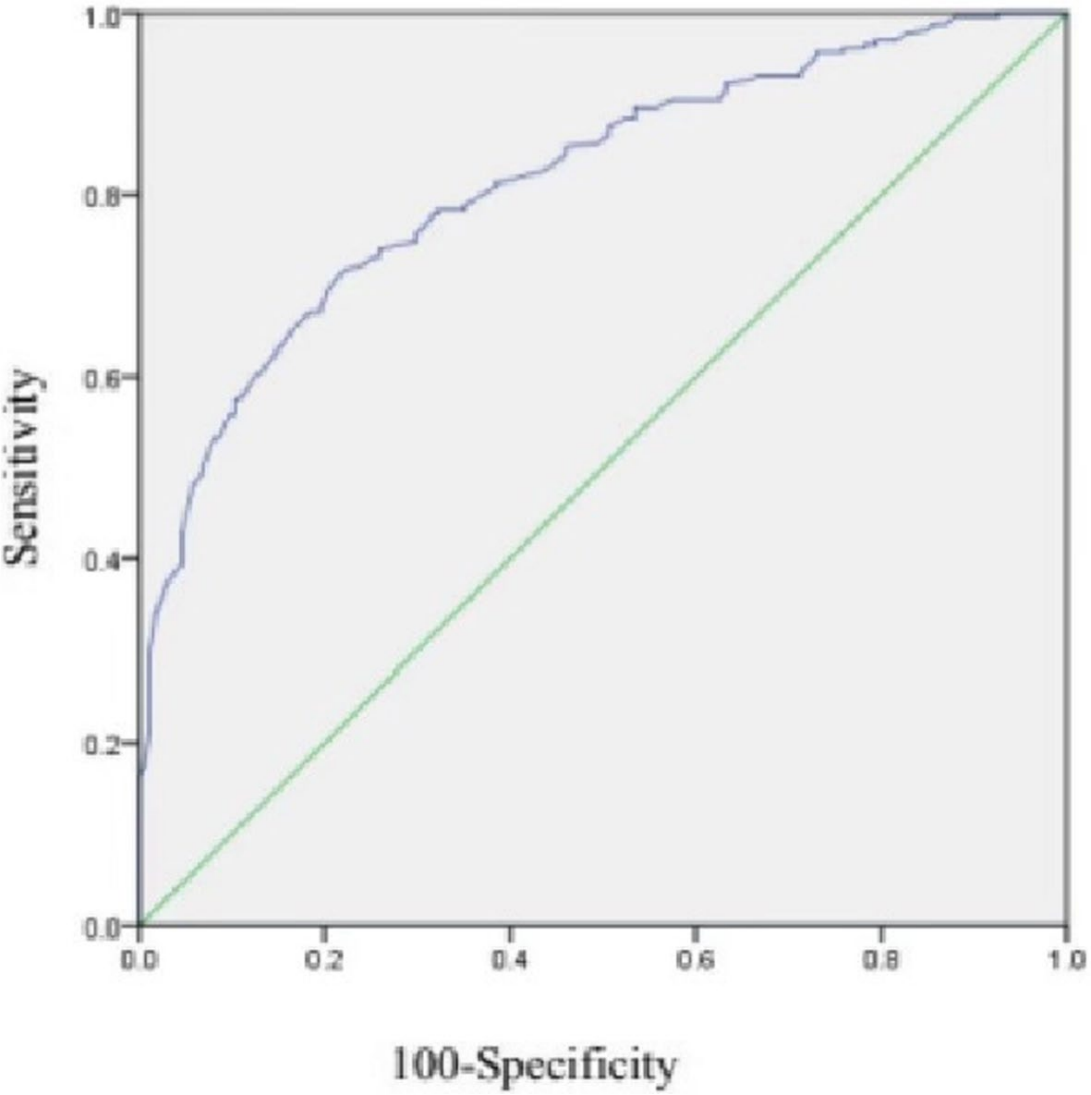
Receiver operating characteristic (ROC) curve of population with MCL of 26-35 days.The area under the TD curve is 0.836. 95% CI 0.775–0.856

ROC curves (Figs. 2 and 3) were plotted in the case and control groups with whether the number of oocytes retrieved reached 15 as the status variable and the AMH value as the test variable. ROC analysis showed that the AMH cutoffs to predict the retrieval of at least 15 oocytes were 3.5 ng/ml and 2.7 ng/ml in the case and control groups, respectively, and the AUC values for these cutoffs were 0.866 and 0.836, respectively. In the case and control groups, the AMH levels were less than 3.5 ng/ml and 2.7 ng/ml, respectively, and the number of oocytes retrieved was less than 15, indicating the onset of a decreased ovarian response.

## Discussion

Compared with AMH, AFC and other indicators to evaluate ovarian function, MCL is easy and inexpensive to obtain and is reliable. Our study suggests that the MCL characteristics of young women are associated with ovarian function and reproductive ability. Compared with individuals with an MCL of 26–35 days, individuals with an MCL of 21–25 days had lower AMH values and fewer retrieved oocytes. In mouse experiments, researchers found that as AMH expression decreased, apoptotic granulosa cells increased, suggesting a positive correlation between AMH and the number of granulosa cells [18]. Studies have shown that AMH can increase the expression of stem cell factor/kit ligand [28] and can regulate the proliferation of granulosa cells in a phased manner to promote the growth of early follicles [29]. In addition, follicle size is the key indicator for successful follicle development [30], and ultrasound measurement of follicle size is a reliable parameter for making many clinical decisions. The proliferation of granulosa cells plays an important role in the growth of follicles [31]. The thickness of the granulosa cell layer in follicles is constant at approximately 45 ± 10 mm, and the number of granulosa cells differs in follicles with different diameters. A larger follicle volume is results in a larger number of granulosa cells within that follicle [32]. At our reproductive center, when ultrasound indicates three or more follicles are 17 mm in diameter or two or more follicles are 18 mm in diameter in both ovaries, the trigger is applied, and oocytes are retrieved under transvaginal ultrasound guidance 36 h after the trigger. During superovulation, the initial and the total amounts of Gn were higher in patients with an MCL of 21–25 days than in patients with an MCL of 26–35 days (*P* < 0.05). Young women with a short MCL need higher doses of Gn to allow follicles to reach a diameter of 17–18 mm. In summary, we speculate that women with short MCLs have fewer granulosa cells in their follicles. The interaction between granulosa cells and oocytes is necessary for follicular development and the maintenance of normal function. The autocrine and paracrine substances produced by granulosa cells can promote the proliferation of granulosa cells and follicular growth [15]. For example, when 10% of granulosa cells undergo apoptosis, follicles become atretic [33]. The normal expression of oocyte genes is closely related to the proliferation, differentiation and function of granulosa cells [34, 35]. Within a certain scope, more granulosa cells present in the follicle will result in a greater capacity for the follicle to develop oocytes [20, 36], and a more satisfactory number of oocytes will be retrieved. Correspondingly, women with an MCL of 21–25 days tend to have fewer oocytes retrieved than women with an MCL of 26–35 days.

The purpose of Gn treatment is to prompt ovarian stimulation, which is the basis of infertility treatment, it induces the development of multiple follicles and oocyte maturation to increase the possibility of conception [32]. The effect of stimulation is largely dependent on ovarian response, and most of the previous criteria for judging ovarian response have included a cutoff for “poor” ovarian response. According to the Patient-Oriented Strategies Encompassing IndividualizeD Oocyte Number (POSEIDON), the AMH cutoff corresponding to poor preinduction ovarian parameters is 1.2 (in a study population of women < 35 years old) [37]. With IVF, when AMH is less than 1.1 ng/ml, the continuation of IVF treatment is no longer recommended because of poor prognosis. Therefore, in most cases, patients under 35 years of age with AMH levels that meet the POSEIDON cutoff are likely to have an unsatisfactory outcome. In this study, we tried to provide AMH cutoffs for “normal” and “weakened” ovarian responses rather than a cutoff for “poor” responses. When patients reach the cutoff of “poor” responses, they are often left with a tighter time frame for treatment, which makes them feel anxious and under greater emotional stress. The success rate in the treatment process is also greatly reduced. However, our cutoffs of “weakened” can serve as an early warning to begin IVF treatment. With a better ovarian response and longer treatment time, not only does it put patients in a better psychological state and reduce anxiety during treatment, but also greatly improve the success rate of treatment and spend less. This has been of great benefit to patients. Before this study, it was difficult for us to provide a clear definition of “good” versus “bad” when AMH values were near 3 without considering the MCL characteristics of young women. Therefore, it was difficult to prompt the vigilance and attention of physicians and patients in such circumstances, which caused some patients to miss the optimal time to receive assisted reproductive technology, and this may result in increased financial costs and mental stress on the road to fertility in the future. While our study improved these conditions, we used 15 oocytes retrieved as the evaluation criteria for normal ovarian response. According to the ROC analysis, the AMH cutoffs for normal ovarian response were 3.5 ng/ml and 2.7 ng/ml among individuals with an MCL of 21–25 days and those with an MCL of 26–35 days, respectively. Consequently, the values of 3.5 ng/ml and 2.7 ng/ml may have important clinical significance. Among individuals with an MCL of 21–25 days and those with an MCL of 26–35 days, AMH levels < 3.5 ng/ml and < 2.7 ng/ml, respectively, are likely to indicate that the ovarian response has begun to decrease. We hypothesize that a shorter menstrual cycle corresponds to fewer granulosa cells. The AMH value is negatively correlated with granulosa cell apoptosis. Therefore, patients with a shorter MCL may require higher AMH levels to reduce the apoptosis rates of granulosa cells, increase the number of granulosa cells and support follicular growth and oocyte maturation.

We also used 5 oocytes retrieved as an indicator of poor ovarian response [38]. However, for the subset of patients with ≤5 oocytes retrieved in the case and control groups, the difference in AMH, the initial Gn amount and the total Gn amount was not statistically significant. We present the following analysis of this finding: it has been demonstrated that miR-17-3p, miR-17-5p, miR-18a-3p and miR-92a-3p, four mRNAs whose high level expression is associated with a high cell proliferation rate and low cell death, are significantly increased in granulosa cells of patients with poor ovarian reserve (POR). In young patients, the granulosa cell proliferation rate is higher in the population with POR than in the population with normal ovarian reserve (NOR) [39–41]. We hypothesize that this increased proliferation is largely responsible for the nonsignificant differences in AMH, initial Gn amount and total Gn amount between these subsets of our study groups. Some studies have proposed that compared with patients with NOR, patients with POR have significantly increased early, late and total apoptosis [20]. We consider the apoptotic rate to be greater than the proliferation rate, and when the two effects are superimposed, the total number of granulosa cells still declines.

As expected, shorter MCLs were associated with lower AMH levels, which is consistent with previous studies [6, 42]. Age is an important factor that affects granulosa cell apoptosis, ovarian response and MCL characteristics [6, 43, 44] . Previous studies did not limit the age range of their study populations, and therefore, age-related confounding factors may have had an effect on the study results. However, our studies limited the age range of the study population, and we found that this conclusion still held true for a young population, which increases our confidence in the independent effect of MCL on ovarian reserve. Some studies have proposed that the difference between the measured AMH concentration and the expected AMH value based on the corresponding AFC cannot be completely explained by the technical limitations of AFC counting and variations in the AMH analyses used. Antral follicle count (AFC), consist of all ultrasonographically identified and counted antral follicles measuring 2–10 mm in diameter in both ovaries, but whether the follicles that are visualized represent potentially healthy follicles with competent oocytes has not been established [45]. In previous studies and clinical practice, there is considerable difference in the clinical definitions and technical methods used to count and measure follicles [46]. Moreover, the accurate measurement of AFC is more demanding for clinicians. If both AFC and AMH are used to predict the ovarian response, the difference between AFC and AMH may affect the patient’s consultation and patient management [47]. Therefore, we used the number of oocytes retrieved as the criteria to evaluate ovarian response. Similar to the results of previous studies [3, 48], our results support that a shorter MCL corresponds to fewer oocytes retrieved. However, we provided AMH cutoff values for ovarian response assessment to populations who have different MCLs within the normal range according to the number of oocytes retrieved, which has rarely been provided in previously published literature and is of great significance for clinical guidance.

Our study also has some limitations. For example, the menstrual cycle is divided into luteal and follicular phases, and we only analyzed the relationship between AMH and total menstrual cycle length MCL and did not have specific luteal and follicular phase lengths. Additionally, granulosa cells are divided into mural granulosa cells and cumulus granulosa cells. We analyzed only the total number of granulosa cells and did not consider the two types separately. Retrospective studies inherently have some limitations, and this study has done some work to reduce the impact of these limitations during the design, implementation, and analysis. First, in terms of data collection, the chart reviewers had a good medical education background and received standardized and uniform training, which ensured the accuracy and consistency of the data collected. And we implemented blinding of the data collectors during the study to keep them blinded to the purpose of the study and the problem the study was trying to address, reducing information bias. Second, the data included in our study were all objective indicators, which were measured by standard hospital instruments, and highly subjective indicators such as “more AFC” were not included in our study variables, so there was no recall bias in this study, which further reduced information bias. Next, in selecting the study population, we screened strictly according to inclusion and exclusion criteria. A large amount of missing data may lead to incorrect conclusions. We have a high-quality electronic medical record system, and when individual data were missing or ambiguous in electronic cases during data collection, we went through the paper medical records for additions, and only 128 samples out of the final 6246 were excluded due to incomplete data. Our strict case inclusion and exclusion criteria, high sample size, and complete database reduced the selection bias in this study. Furthermore, we performed PSM based on the patient’s age and BMI, thus controlling for confounding by age and BMI confounders, controlling for confounding bias, and improving the efficiency of the study. Some limitations of retrospective studies are difficult to avoid, and we expect better methods and measures to reduce these limitations in the future. Outlook: We conclude that young women with shorter MCLs have fewer granulosa cells and that the number and proliferation of granulosa cells in different groups require further study to extend these important and relevant results. Cumulus granulosa cells and mural granulosa cells are correlated with oocyte maturation, and their gene expression is correlated with embryo quality and live births. Which kind of granulosa cell number has a greater impact on outcome is still unclear to this point, and relevant basic research is needed to verify this conclusion.

## Conclusion

An MCL of 21–25 days has a significant negative correlation with AMH and ovarian response, and populations with these MCLs require an increased dosage of Gn during COS to retrieve more oocytes. AMH levels < 3.5 ng/ml among individuals with an MCL of 21–25 days and < 2.7 ng/ml among individuals with an MCL of 26–35 days population may be cutoffs for the onset of a decline in ovarian response.

## Data Availability

The data used or analyzed during the current study are included within the article. The datasets are not publicly available due to the hospital policy and personal privacy. However, the datasets are available from the corresponding author on reasonable request.

## Abbreviations

IVF: In in Vitro Fertilization
MCL: Menstrual cycle lengths
COS: Controlled ovarian stimulation
GnRH-a: Gonadotrophin-releasing hormone antagonist
POR: Poor Ovarian Response
Gn: Gonadotropin
LH: Luteinizing Hormone
FSH: Follicle Stimulation Hormone
BMI: Body Mass Index
TGF-β: Transforming Growth Factor Beta
AMH: Anti-Müllerian Hormone
P: Progesterone
T: Testosterone
E2: Estradiol
AFC: Antral follicle count
HCG: Human chorionic gonadotropin
ROC: Receiver operating characteristic
AUC: Areas under the curves
NOR: Normal ovarian reserve
PSM: Propensity score matching
TVS: Transvaginal sonography
